# Sway frequencies may predict postural instability in Parkinson’s disease: a novel convolutional neural network approach

**DOI:** 10.1186/s12984-025-01570-7

**Published:** 2025-02-18

**Authors:** David Engel, R. Stefan Greulich, Alberto Parola, Kaleb Vinehout, Justus Student, Josefine Waldthaler, Lars Timmermann, Frank Bremmer

**Affiliations:** 1 Applied Physics and Neurophysics, Philipps-Universität Marburg, Karl-von-Frisch-Straße 8a, Marburg, 35032 Germany; 2 Department of Neurology, Oregon Health & Science University, Portland, OR USA; 3 Center for Mind, Brain and Behavior (CMBB), Philipps-Universität Marburg, Marburg, Germany; 4 Chair of Business Information Systems, Esp. Intelligent Systems and Services, TUD Dresden University of Technology, Dresden, Germany; 5 Centre for Language Technology, Department of Nordic Studies and Linguistics , Copenhagen University, Copenhagen, Denmark; 6 Cold Spring Harbor Laboratory (CSHL), Cold Spring Harbor, NY USA; 7 Department of Neurology, University Hospital Giessen and Marburg, Marburg, Germany

**Keywords:** Parkinson’s disease, Postural instability, Body sway, Center of pressure, Center of mass, Frequency analysis, Deep learning, Convolutional neural network

## Abstract

**Background:**

Postural instability greatly reduces quality of life in people with Parkinson’s disease (PD). Early and objective detection of postural impairments is crucial to facilitate interventions. Our aim was to use a convolutional neural network (CNN) to differentiate people with early to mid-stage PD from healthy age-matched individuals based on spectrogram images obtained from their body sway. We hypothesized the time–frequency content of body sway to be predictive of PD, even when impairments are not yet clinically apparent.

**Methods:**

18 people with idiopathic PD and 15 healthy controls (HC) participated in the study. We tracked participants’ center of pressure (COP) using a Wii Balance Board and their full-body motion using a Microsoft Kinect, out of which we calculated the trajectory of their center of mass (COM). We used 30 s-snippets of motion data from which we acquired wavelet-based time–frequency spectrograms that were fed into a custom-built CNN as labeled images. We used binary classification to have the network differentiate between individuals with PD and controls (*n* = 15, respectively).

**Results:**

Classification performance was best when the medio-lateral motion of the COM was considered. Here, our network reached a predictive accuracy, sensitivity, specificity, precision and F1-score of 100%, respectively, with a receiver operating characteristic area under the curve of 1.0. Moreover, an explainable AI approach revealed high frequencies in the postural sway data to be most distinct between both groups.

**Conclusion:**

Heeding our small and heterogeneous sample, our findings suggest a CNN classifier based on cost-effective and conveniently obtainable posturographic data to be a promising approach to detect postural impairments in early to mid-stage PD and to gain novel insight into the subtle characteristics of impairments at this stage of the disease.

## Background

Parkinson’s disease (PD) currently affects around 1% of the population over 60 [1, 2] and is predicted to affect around 3% of the world population over 65 by 2030 [3]. Postural instability is considered one of the most disabling features of PD and at later stages increases the risk of falls and significantly reduces quality of life [4–10].

The gold standard to measure postural instability in clinical settings consists of the Movement Disorder Society Unified Parkinson’s Disease Rating Scale (MDS-UPDRS) [11–13] and the Hoehn and Yahr scale [14], performed by clinicians. Even though scales like the MDS-UPDRS score have been attested a good test–retest reliability [11], most clinical balance ratings remain (semi-)subjective, have been shown to be incapable of detecting mild changes [15], and often show poor sensitivity and inconsistency between trials [16, 17]. In addition, motor symptoms visible to a clinician during these tests typically occur at later stages of the disease, while it has been suggested that postural impairments might already be present in the early motor phase and that they might bear the potential as a pre-diagnostic tool to detect the disease [18–21]. While state-of-the-art symptomatic treatments with levodopa or deep brain stimulation (DBS) may not help postural stability [22–25], objective detection of subtle balance impairments might allow for timely countermeasures, as there is evidence for positive effects of exercise and specific balance training on postural control [26].

Hence, there is a strong need for objective and easy to obtain measures of postural control to assist clinical testing of PD [3, 27]. Many studies have been trying to find objective markers of postural sway during quiet stance that distinguish individuals with PD from healthy controls (HC) [28–31]. Most of these studies tried to obtain these markers based on traditional spatiotemporal sway parameters such as sway amplitude, path length, area, and velocity, but some have also explored frequency domain measures like the centroidal frequency or frequency dispersion of sway [32–37]. Nonetheless, results thus far have been contradictory, which has mainly been attributed to the wide heterogeneity of affected individuals and wide variety of study designs (for a review, see [38]). In addition, all these measures are usually acquired in laboratory experiments which often require expensive hardware and trained experts performing elaborate protocols, resources most often not available to medical practitioners, especially in developing countries [39]. Taken together, currently available objective measures thus far yield inconsistent results and are often cumbersome to assess, which is why a high-performing approach based on easily and quickly obtainable data is needed.

As in many other fields, machine learning techniques are being increasingly used to discriminate between healthy individuals and people with PD. In a recent review of 209 studies within the machine learning literature, prediction accuracies between 80 and 100% have been found using data such as voice recordings, gait patterns, hand writings and MRI imaging [40]. In terms of static balance assessments, different machine learning algorithms have been compared on various features of center of pressure (COP) sway recorded during quiet standing, leading to prediction accuracies between 64 and 83.9% [41, 42]. Some recent studies have also used deep learning approaches, where computational models are composed of multiple processing layers to learn representations of data with multiple levels of abstraction [43]. Convolutional neural networks (CNN) constitute a widely established computer vision algorithm for image classification, with an architecture somewhat similar to the ventral stream of the human visual cortical system and consist of various layers that use learnable filters for feature extraction [43, 44]. CNN approaches have been used in the context of PD based on brain imaging data like SPECT, MRI, and PET, but also successfully on physiological data like gait patterns, handwritings, and speech [45].

Given these promising applications of machine learning methods to various aspects of the disease, the aim of our study was to develop a CNN classifier trained on posturographic data based on a quick and easy assessment. For this purpose, we recorded the COP and center of mass (COM) trajectories of individuals with PD and age-matched HC during quiet standing using mobile and cost-effective devices such as a Wii balance board and a Kinect motion sensor [46–48] and trained a custom-built CNN to distinguish both groups based on the frequency content of their body sway. We hypothesized that a CNN trained on time–frequency spectrogram images acquired during quiet stance can reliably distinguish individuals with early to mid-stage PD from age-matched HC subjects and thus bears great potential not only for future clinical applications, but also for a better understanding of standing posture in PD.

## Methods

### Participants

We assessed a group of 18 individuals with idiopathic PD (age: range: [42–75]; mean ± standard deviation: 58.10 ± 8.66) in early to moderate disease stages (Hoehn and Yahr: ([1–3]; 1.94 ± 0.70) [14] with a mean disease duration of 4.8 years ([0–15]; 4.79 ± 4.71)) who were diagnosed according to the Movement Disorder Society diagnostic criteria [49]. To be included into the study, individuals with PD had to be able to walk without any assistance and not have more than one reported fall in the year prior to the study. Also, no *Freezing of Gait* was to occur neither in the preliminary clinical examinations nor during the measurement. All individuals with PD were measured while on their regular dose of dopaminergic medication (levodopa equivalent daily dose (LEDD): [105-1980]; 651.63 ± 529.97). Detailed information on the individuals recruited for our PD group can be found in Table 1. For the control group (healthy controls, HC), we recruited fifteen age-matched healthy adults (age: [49–69]; 59.80 ± 6.45). General exclusion criteria were any existing neurological or psychiatric (e.g., neuropathies, epilepsy, multiple sclerosis, schizophrenia, severe depression, dementia, etc.) disorders or orthopedic conditions that might affect upright stance and balance control (e.g., hip, spine, knee, etc.). Potential cognitive impairment was evaluated prior to the study based on the Montreal Cognitive Assessment with a cut-off score of 24 points [50]. All subjects had normal or corrected to normal vision.

**Table 1:** Participant demographics along with clinical scores of the individuals recruited for the PD group

ID	Age	MoCA score	Disease duration (months)	H&Y stage	MDS-UPDRS III (ON medication)	LEDD (mg)
PD 1	55	27	79	2	40	900
PD 2	51	30	29	1	2	105
PD 3	70	26	9	2	16	282
PD 4	52	29	5	2	5	250
PD 5	51	29	15	1	9	242
PD 6	62	26	86	3	66	821
PD 7	53	29	176	2	18	935
PD 8	70	28	40	3	n.a	210
PD 9	50	29	62	3	21	774
PD 10	55	28	21	1	20	150
PD 11	53	28	43	2	11	786
PD 12	68	26	188	3	n.a	1880
PD 13	53	29	133	2	20	1980
PD 14	76	24	32	2	n.a	465
PD 15	65	28	61	1	n.a	515
PD 16	57	29	32	2	25	780
PD 17	42	29	2	1	9	121
PD 18	63	28	33	2	46	532
HC 1	53	27	–	–	–	–
HC 2	50	30	–	–	–	–
HC 3	66	30	–	–	–	–
HC 4	65	30	–	–	–	–
HC 5	60	29	–	–	–	–
HC 6	70	29	–	–	–	–
HC 7	53	30	–	–	–	–
HC 8	53	30	–	–	–	–
HC 9	61	29	–	–	–	–
HC 10	59	29	–	–	–	–
HC 11	62	30	–	–	–	–
HC 12	49	28	–	–	–	–
HC 13	65	26	–	–	–	–
HC 14	67	30	–	–	–	–
HC 15	64	29	–	–	–	–

*MoCA* Montreal Cognitive Assessment, *H&Y* Hoehn & Yahr, *MDS-UPDRS III* total score obtained from Part III of the Movement Disorder Society Unified Parkinson’s Disease Rating Scale, *LEDD* levodopa equivalent daily dose, *n.a.* no data available

### Experimental setup

Experimental data for this study was acquired as part of two previous studies [47, 48]. Participants stood on a Wii Balance Board (WiiBB, Nintendo, Kyoto, Japan) to track their COP. Wearing no shoes, they were instructed to position their feet about shoulder width apart, about parallel on the ground. In one experimental condition, participants were to stand quietly with eyes open in a virtual 3-D environment, which consisted of a tunnel stretching in the anterior–posterior direction. During trials, they were instructed to stand relaxed with their arms hanging loosely at their sides. They had to fixate a target in the center of the far end of the tunnel to ensure their gaze remained straight ahead. In this manner, this condition simulated quiet standing in a natural environment with eyes open. One trial of measurement lasted for 30 s and was preceded and followed by resting periods where participants could relax and stretch as long as they needed. Each participant performed a total of 10 trials. To track their body motion, we used a Kinect v2 video-based motion tracking system (Microsoft, Redmond, WA, USA) which recorded the 3D-positions of 25 different ‘body joints’ as determined by an internal algorithm. The camera was located 210 cm in front of the participants and fixed at a height of 140 cm. The visual environment was presented through a head-mounted virtual reality headset (HTC Vive, HTC, New Taipei City, Taiwan). The frame rate was 90 Hz. The field of view extended over 110° in the vertical as well as in horizontal direction. Participants were secured by a harness which was attached to the ceiling. We ensured that the harness guaranteed subjects’ safety but was not providing lift during trials. For a more detailed description and depictions of the technical setup and experimental protocol, please refer to our previous work [47, 48].

### Data processing, architecture, and evaluation

**Fig. 1:**
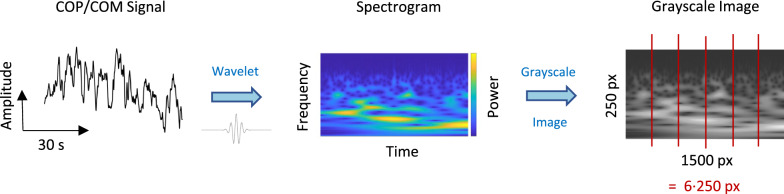
Data preprocessing. The time-course signals of the COP and COM trajectories underwent wavelet decomposition, resulting in spectrogram images with frequency power as a function of time and frequency. These spectrograms were then treated as grayscale images. Subsequently, each image was cut into six sections. The final samples constituted grayscale images with a size of 250 × 250 px

Out of the WiiBB sensor data, we calculated the anterior–posterior (a–p) and medio-lateral (m-l) COP trajectories for each trial. The respective trajectories of the center of mass (COM) from the Kinect data were obtained based on the interpolated center positions of relevant body segments along with their attributed mass contributions, which were taken from anthropometric tables [51]. Hence, our spatiotemporal data set consisted of time series of two postural parameters, the COP and COM, with two spatial directions per parameter, the a–p and m-l directions of body sway, respectively. Data of each parameter and direction was detrended (subtraction of the mean) and resampled to 50 Hz using a custom-written Gaussian moving average filter with a symmetric window (sigma = 1/60 s), resulting in 1500 time points per 30 s-trial. We then performed a wavelet decomposition (generalized Morse wavelets, gamma: 3; 36 voices per octave; frequency range: [0.12–15 Hz]) on all trajectory data to acquire time–frequency spectrograms for each trial. Subsequently, to obtain more data samples, the spectrograms were cut into 5 s-segments (250 time points), resulting in a total of 60 wavelet-based time–frequency spectrograms per subject (composed of 10 trials * 6 time segments) for each parameter and direction. Each spectrogram contained 2-D data (250 time points * 250 frequency bands) with one energy value associated with each time–frequency point, representing the respective frequency power. The resulting spectrograms thus resembled 250 × 250 px grayscale images with the energy values represented as pixel values (Fig. 1).

**Fig. 2:**
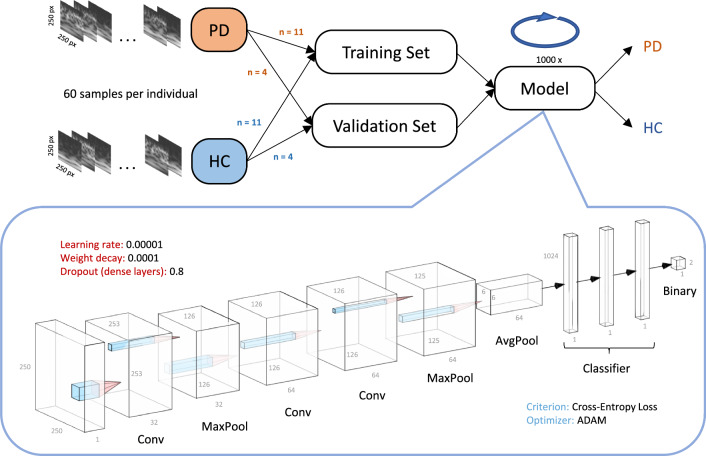
Data pipeline and architecture. Data was split into train and validation sets on a randomized per-subject basis. For each model that we trained, data from 11 participants out of each group, consisting of the corresponding 60 sample images per participant, went into the training set. The remaining data went into the validation set. The model was then trained and evaluated for 1000 epochs. We built a CNN with three convolutional and three pooling layers, feeding into a classifier network consisting of three fully-connected layers. Network architecture was visualized using NN-SVG [52]

Network training using the previously acquired spectrogram images was performed for each parameter (COP, COM) and direction (a-l, m-l) separately. Since one of our aims was to establish a system that can classify new subjects, the spectrograms from each group were split into train and validation sets on a per-subject basis. Hence, all 60 spectrogram images of each participant were assigned either entirely to the train or validation set, respectively. Due to the Kinect occasionally losing tracking, 10% of the total trials in the PD group and 3% of the total trials in the HC group needed to be discarded, resulting in some participants providing less than 60 samples (PD: *n* = 7, HC: *n* = 3). To avoid biases due to unequal group sizes, for each model we trained, only 15 out of the 18 subjects with PD were randomly selected, resulting in 15 subjects in both groups. Subject data was labeled binarily regarding group. Subsequently, 11 subjects (660 samples) from each group were assigned to the training set, while the remaining 4 subjects (240 samples) were assigned to the validation set (Fig. 2).

Once assigned to the train and validation sets, the pixel values of the spectrograms were normalized according to mean and standard deviation across all subjects. Importantly, the normalization coefficients obtained from the training set were applied to both train and validation set.

We built a custom CNN using the PyTorch framework [53]. The feature extractor network consisted of three convolutional and three pooling layers, the classifier consisted of three dense layers ending in a binary classification between PD and HC (Fig. 2). Each model was trained on full data batches over 1000 epochs with a learning rate of 0.0001 and slight regularization (weight decay = 0.001). We used an ADAM optimizer with cross-entropy-loss. A total of 250 models were trained for each parameter (COP, COM) and direction (a–p, m-l). For cross-validation purposes and to facilitate generalizability, i.e., to prevent overfitting on a specific subset of participants, the per-subject assignment to each respective train and validations set was randomized before a new model was trained, i.e., each model was trained and tested on data based on different participants. In the end, we evaluated all 250 models on average validation set performance for each parameter and direction. Evaluation metrics were predictive accuracy, sensitivity, specificity, precision and F1-score on validation set data as well as corresponding receiver operating characteristics (ROC) curves and precision-recall (PR) curves with area under the curve (AUC) [54]. We also created a baseline performance to check whether the actual network performance was based on a true difference in the spectrograms between the groups. For this purpose, we also trained 250 models with randomly assigned labels and performed the same evaluation as with the correctly labeled data sets. Network performance was compared with the baseline performance using t-tests on the validation set accuracy after 1000 epochs of training across all 250 models, respectively. We considered a *p* value <0.05 to reject the hypothesis that model performances between the shuffled and actual data sets came from the same distribution.

To gain insight into the decision process of the trained models, we employed an explainable AI approach utilizing Gradient-weighted Class Activation Mapping (GradCAM, [55]). GradCAMs identify the gradient information flow into the decision layer for the decoded class from each pixel of the input. The output can be visualized by heatmaps of the same size as the input image. These heatmaps indicate areas on which the model is focusing for classification, identifying which parts of the input image contribute the most to the class decision in the decision layer.

## Results

### Excellent classification performance in discriminating people with PD from healthy controls

**Fig. 3:**
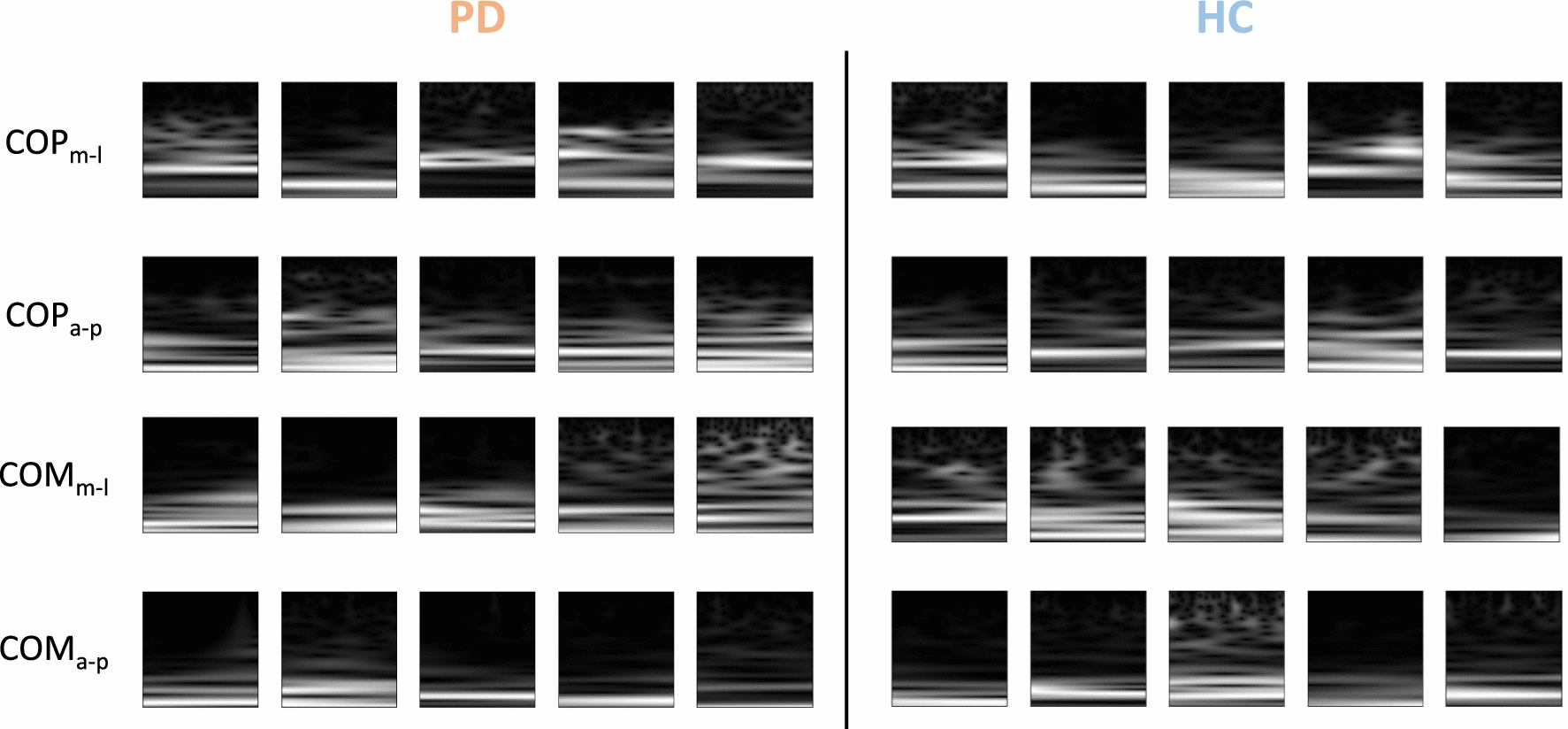
Sample grayscale spectrogram images from randomly picked participants as they are fed into the CNN. Rows represent each postural parameter we investigated. Left side shows spectrograms obtained from individuals with Parkinson’s disease (PD), right side shows spectrograms from healthy controls (HC). COP: center of pressure, COM: center of mass, m-l: medio-lateral, a–p: anterior–posterior

Figure 3 shows randomly picked sample spectrogram images obtained through wavelet decomposition from the anterior–posterior and medio-lateral COP and COM signals. Some participants expressed a lot of energy in the lower frequency bands of their sway (bright pixels in the lower sections of the image) while higher frequencies were suppressed (dark pixels in the upper sections of the image). Others expressed more equally dispersed energy across all frequency bands. In general, similar samples could be found across all parameters and in both groups, preventing discriminability by the naked eye.

Table 2 displays the prediction performance of our network for the COP and COM measures in the anterior–posterior (a–p) and medio-lateral (m-l) directions, respectively, averaged over 250 training sessions with randomized train- and validation set assignments of individual subjects (see Methods). When trained on COP data, average validation accuracy, sensitivity, specificity, precision and F1-score reached about 99%, 99%, 99%, 100% and 99% for the m-l direction, respectively. Training on a–p COP data led to accuracy, sensitivity, specificity, precision and F1-score of around 90%, 85%, 89%, 92% and 87%, respectively. Also, results were more stable for the m-l direction (standard deviations of 4.5–7.9% vs. 14.8–25.5% for a–p). The receiver operating characteristics area under the curve (ROC AUC) and precision-recall area under the curve (PR AUC) reached values of 99.5% and 98.7% for the m-l direction and 92.2% and 91.4% for the a–p direction, respectively (Table 2).

**Table 2:** CNN performance on data from each postural parameter and corresponding direction

Performance measure (% average ± SD)	Accuracy	Sensitivity	Specificity	Precision	F1-score	ROC AUC	PR AUC
COP_m-l_	99.21 ± 04.47	98.55 ± 07.91	98.93 ± 05.50	99.75 ± 02.06	98.99 ± 05.79	99.54 ± 03.79	98.68 ± 03.08
COP_a–p_	89.73 ± 16.90	85.25 ± 25.55	89.22 ± 17.83	91.78 ± 15.09	87.25 ± 21.90	92.17 ± 14.84	91.36 ± 14.84
COM_m-l_	100.0 ± 0.0	100.0 ± 0.0	100.0 ± 0.0	100.0 ± 0.0	100.0 ± 0.0	100.0 ± 0.0	97.86 ± 03.35
COM_a–p_	98.08 ± 08.19	97.34 ± 12.27	98.09 ± 08.12	98.07 ± 09.44	97.54 ± 11.21	98.46 ± 08.24	97.64 ± 07.83
COM_m-l_ (shuffled)	50.03 ± 12.24	51.58 ± 35.01	58.02 ± 22.47	44.06 ± 19.79	46.75 ± 22.30	49.58 ± 19.85	48.10 ± 13.07

Data based on 250 models, each trained with data from different subjects in the train and validation sets

*m-l* medio-lateral, *a–p* anterior–posterior, *ROC AUC* receiver operating characteristics area under the curve, *PR AUC* precision-recall area under the curve

The network performed even better when trained on the COM data. Here, average performance scores reached about 98% accuracy, 97% sensitivity, 98% specificity, 98% precision, 98% F-1 score, 98% ROC AUC and 98% PR AUC for the a–p direction. When trained on the COM data in m-l direction, the network’s performance reached 100% accuracy, 100% sensitivity, 100% specificity, 100% precision, 100% F-1 score, 100% ROC AUC and 98% PR AUC. This demonstrated excellent predictive performance which proved to be consistent across training sessions, indicated by zero variability in almost every score across the 250 models. To ascertain that our network’s performance was based on an actual difference in the data between both groups, we created an additional data set based on the COM data in m-l direction. Here, we shuffled the labels (PD vs. HC) assigned to each sample and trained another 250 models, again each time with random assignment of participants to the train and validation set. Even though with high variability (standard deviations of 12.2–35.0%), in this case, all performance scores remained around chance level (50%) (Table 2, bottom row).

**Fig. 4:**
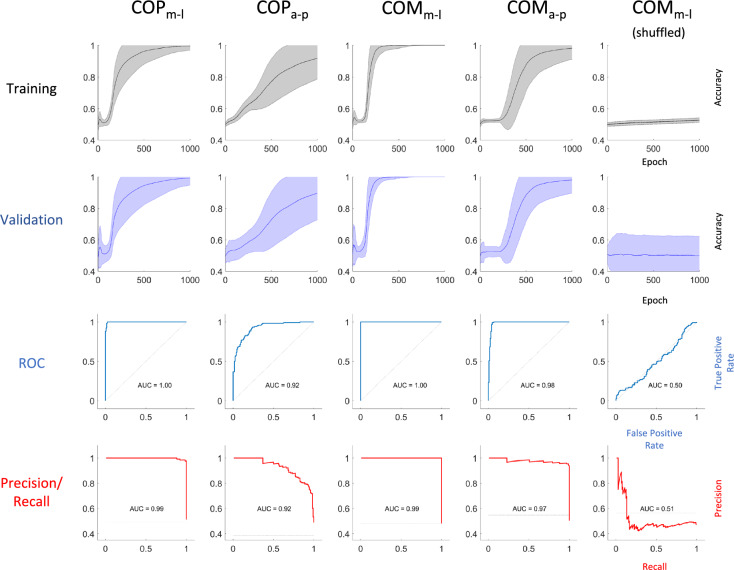
Detailed performance on all parameters and directions. First and second row: Network performance indicated by classification accuracy evaluated on the train and validation set over 1000 epochs of training. Solid lines represent the average across 250 models with different subjects assigned to the train and validation sets in each case. Shaded areas indicate standard error. Third row: Representative ROC curves evaluated on validation data after 1000 epochs of training. Fourth row: Representative precision-recall curves evaluated on validation data after 1000 epochs of training. Rightmost column shows results from 250 models trained on medio-lateral COM data with randomized labels

Figure 4 shows detailed performance measures for all parameters and directions. Accuracy on the training set increased monotonously over the course of 1000 epochs in each case (first row). For both the COP and the COM, performance was best when trained on m-l data. Here, training and validation accuracy quickly ramped up and plateaued at high values, most prominently for the COM data. Our network’s performance was almost identical on the validation sets, i.e., on images that it has not seen during training (Fig. 4, second row). This indicates that it performed as well on novel data as it did on data it has been trained with. The third and fourth rows of Fig. 4 show the receiver operating characteristic and precision-recall curves of representative models which performed close to the average for each parameter and direction. Our network was able to reach excellent sensitivity and specificity for almost all parameters and directions. In general, performance was better for the m-l direction vs. the a–p direction and for COM data vs. COP data.

The rightmost column of Fig. 4 shows performance on the COM data in m-l direction with shuffled labels. Without accurate label information, validation accuracy after 1000 epochs of training remained at chance level, confirmed by the ROC and precision-recall curves. There was a significant difference in validation accuracy across 250 models between the actual and shuffled COM m-l data sets (*p* < 0.0001).

### Frequency content above 1 Hz was most influential to the network’s decision process

**Fig. 5:**
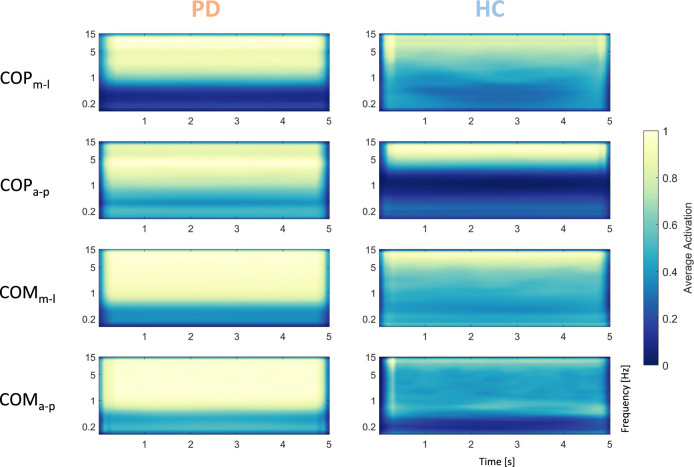
Average gradient class activation maps (GradCAM) based on the last convolutional layer of one representative model for each parameter and direction. Left panels show average GradCAMs for individuals with PD, right panels show GradCAMs for HC. Large weights were given to pixels above 1 Hz when an individual with PD was classified. GradCAMs based on classification of HC were more varied, but also pointed towards high frequencies

We calculated GradCAMs based on one representative model for each parameter and direction. For this purpose, we used the complete batch of validation set images and averaged them over each decoded class. The results can be seen in Fig. 5. For all parameters, pixels representing high frequency bands above 1 Hz were given the largest weights in cases where an individual with PD was classified (Fig. 5, left column). GradCAMs for classification of HC looked more varied, but also pointed towards higher frequencies (Fig. 5, right column). In general, the medio-lateral direction produced more consistent GradCAMs across models, whereas GradCAMs for the anterior–posterior sway direction were more varied. Noteworthy, weights remained consistent over time in all models.

## Discussion

In this study, we developed a convolutional neural network classifier based on posturographic data which we obtained from individuals with PD and healthy age-matched control participants using mobile and cost-effective equipment. Our classifier was trained to distinguish between individuals of both groups based on the time–frequency content of their COP and COM trajectories during quiet stance, represented as spectrogram images. Moreover, we used GradCAMs as an explainable AI approach to investigate which sections of the spectrograms were vital for each model’s decision. We found our network to reach great classification performance in discriminating between individuals with PD and healthy controls on all postural parameters and directions, with exceptional results for medio-lateral body sway data obtained from their COM (Table 2). In general, within each parameter, all performance metrics we evaluated exhibited about equal scores, suggesting balanced classification performance across classes. In each parameter and direction, the largest variability between models occurred in the sensitivity scores, which indicated that our models were more likely to misclassify an individual with PD as healthy (false negative) than identifying a HC as PD (false positive). This variability, however, did not occur in case of the medio-lateral COM data. Here, all models exhibited 100% accuracy, sensitivity, specificity, precision and F1-score, respectively. This means, independent of which participants were selected for the train and validation sets, all samples of the validation set were correctly classified. The results from our dataset with shuffled labels confirm that our models’ classification performance stems from an actual difference between the groups. This made us confirm our general hypothesis that there are differences in the frequency content of body sway signals between both groups that are reliably detectable with a computer vision approach. Applying the GradCAM technique allowed us to obtain interpretable insight into the network’s decision process by revealing that frequency bands above 1 Hz were given the highest emphasis in all parameters and directions. However, this general trend was only consistent across models trained on the medio-lateral direction of both the COP and COM. When comparing models trained on the anterior–posterior direction, GradCAMs appeared more varied, with some models also emphasizing lower frequencies. This might be due to the explainability of GradCAMs being noisy [56] but could also stem from physiological differences in sway between the medio-lateral and anterior–posterior direction.

The fact that the frequency content of COM motion was in general more predictive of PD than that of the COP (Table 2) might stem from the different aspects of postural control these two measures are claimed to reflect [57]. The COM is generally considered the ‘controlled variable’ and its position and motion represent the total outcome of sensorimotor processing by the central nervous system (CNS) when maintaining balance [58, 59]. On the contrary, position and motion of the COP more likely reflect the musculoskeletal response or ‘effort’ of the CNS to control the position of the COM, as the COP tracks the COM and represents the forces enacted on the ground to maintain and restore upright posture [60]. However, classification performance was almost as good on the COP data, which indicates that in terms of frequencies during unperturbed quiet stance, individuals with PD show deviations in both their general ability to maintain an upright posture and in in their effort to control it.

The finding that our network performed better on data obtained from medio-lateral body sway appears plausible as PD motor symptoms are lateralized, especially in early disease stages [61, 62]. This finding indicates a larger difference in the time–frequency spectrograms between the groups and confirms various studies in the literature that used traditional sway parameters in the spatiotemporal realm. For instance, it has been found that individuals with PD show increased postural sway and stochastic activity in their medio-lateral postural sway in static balance assessments [18, 29, 63–65], which lead to the suggestion that lateral instability may be an important posturographic marker of functional balance impairment in PD [63, 65, 66]. This has been confirmed in a recent study using spatiotemporal analyses, where it was found that investigation of the medio-lateral direction of postural sway was best suited to differentiate between individuals with PD and HC [67]. Medio-lateral instability has also been found to better predict fall risk in PD [68]. Moreover, as all people with PD who participated in our study received levodopa at the time of testing, there is evidence that levodopa has negative effects on medio-lateral stability more so than on anterior–posterior stability, possibly increasing distinguishability for our deep learning models [22, 35].

Our GradCAMs (Fig. 5) suggest a common postural trait among the individuals with PD that manifests in higher frequency components of their postural sway during quiet stance. Biomechanically, higher frequencies have been associated with increased stiffness around the ankle joint [69], that might be explained by the increased rigidity commonly associated with PD [18, 47]. In terms of central-nervous processing of balance control, three frequency bands are usually associated with involvement of specific neuronal loops: 0–0.5 Hz for visuo-vestibular regulation, 0.5–2 Hz for cerebellar participation, and 2–20 Hz for proprioceptive participation [42, 70]. Since our models detected differences between individuals with PD and HC mostly in the frequency bands above 1 Hz, this might reflect impairments in the latter two central-nervous loops: The lower range of the most distinctive frequency bands might represent altered cerebellar activity in PD, which has been suggested to be pathological or compensatory [71, 72]. The higher range of the most distinctive frequency bands, on the other hand, might reflect impaired proprioceptive processing, which is also commonly associated with PD [4, 73, 74].

Lastly, the frequency bands revealed by our GradCAMs overlap with those of the slow resting-tremor commonly associated with PD, which manifests between 4 and 7 Hz [75]. However, pixels way above those frequencies, exceeding 10 Hz and beyond, were given similarly large weights in our network’s decisions. Moreover, we checked for tremor frequencies in the hand motion of all participants. This revealed that two of the individuals with PD exhibited a tremor, one of them in only one hand. In both cases, the tremor frequency was around 5 Hz, as reported in the literature. Thus, since only two out of 18 participants with PD exhibited a tremor, we exclude tremor as potential cause for the high gradients in the individuals with PD. This suggests the selectivity for those frequency bands and above to stem from postural motion. Another trait that is shared between all GradCAMs constitutes the weight consistency across time. On average, there seems to be no relevant temporal information in the spectrograms for the network’s decision process. On the physiological side, this means that the frequency content of postural sway during quiet stance seems to remain stable over time, for individuals with PD and healthy adults alike. In terms of classification, in our case, this means that simpler classifiers trained on 1-D frequency data might be sufficient, with the additional advantage of even larger sample sizes, since singular time points could be used.

Despite our promising results, we want to acknowledge that our study has several limitations and consider it to be of pilot nature. Even though the heterogeneity in our group of people with PD somewhat reflected the general population with early to mid-stage PD, the high variabilities in age, disease duration and medication dosage (Table 1) likely undermine the generalizability of our findings. Even though progression is often not linear, older age and longer disease durations are usually associated with more severe motor impairments and vice versa [76]. Similarly, higher dosages of dopaminergic medication likely produce more pronounced motor improvements, even though patients generally receive a medication dose that provides optimal symptom control on an individual basis, and studies on the effect of medication on static balance remain inconclusive [19, 22, 24, 25, 35, 77]. These variabilities are also reflected in the large spread of UPDRS Part III-scores in our PD group. They represent clinical assessment of motor impairments at the time of testing and in our group range from 2 (very mild) to 66 (severe). Furthermore, the main limitation of our study marks the small number of participants in the context of a deep learning approach. Even though our robust performance metrices and consistency in the GradCAMs suggest that our methods were able to capture common traits that were unique to people with PD, and we randomized data into the respective train and validation set of each model and cross-validated across many models, we cannot exclude the possibility that our models overfitted to our specific data set. Moreover, due to the smaller pool of participants, there was no variability in data from the HC group between different models. Thus, expanding our study to include a larger and more heterogeneous sample would be essential for improving the reliability and clinical applicability of our findings. In particular, it would be intriguing if our approach was able to find differences in people with de-novo PD who have not started any Parkinsonian medication yet, as this would mark a large step towards assisting clinicians in early detection and intervention, since timely neuroprotective and neuromodulatory therapies may have the potential to delay disease progression at an early stage [78, 79].

## Conclusions

We trained a convolutional neural network to classify individuals with early to mid-stage PD based on the frequency content of their body sway during quiet standing and achieved excellent classification performance, suggesting that postural impairments might be reflected in specific frequency bands. Moreover, our explainable AI approach (GradCAM) might provide meaningful insight into posturographic data obtained from a PD population. As our results can be achieved with short recording times and minimal experimental effort, our study design can easily and conveniently be applied in clinical settings and bears potential to deepen understanding of postural instability in PD and to facilitate clinical evaluation of the disease. However, given the small and heterogeneous group of participants, our findings are of preliminary nature and need to be confirmed with a larger and more heterogeneous sample.

## Data Availability

The datasets analyzed in this study are available as open-access zenodo repository: 10.5281/zenodo.13277579.

## Abbreviations

PD: Parkinson’s disease
CNN: Convolutional neural network
HC: Healthy controls
COP: Center of pressure
COM: Center of mass
MDS-UPDRS: Movement Disorder Society Unified Parkinson’s Disease Rating Scale
DBS: Deep brain stimulation
LEDD: Levodopa equivalent daily dose
WiiBB: Wii Balance Board
a–p: Anterior–posterior
m-l: Medio-lateral
ROC: Receiver operating characteristics
PR: Precision-recall
AUC: Area under the curve
GradCAM: Gradient-weighted class activation map
SD: Standard deviation
CNS: Central nervous system

## References

[1] Elbaz A, Carcaillon L, Kab S, Moisan F. Epidemiology of Parkinson’s disease. Rev Neurol. 2016;172:14–26. 10.1016/j.neurol.2015.09.012. 26718594 10.1016/j.neurol.2015.09.012

[2] Tysnes O-B, Storstein A. Epidemiology of Parkinson’s disease. J Neural Transm. 2017;124:901–5. 10.1007/s00702-017-1686-y. 28150045 10.1007/s00702-017-1686-y

[3] Palakurthi B, Burugupally SP. Postural instability in Parkinson’s disease: a review. Brain Sci. 2019;9:239. 10.3390/brainsci9090239. 31540441 10.3390/brainsci9090239 PMC6770017

[4] Benatru I, Vaugoyeau M, Azulay JP. Postural disorders in Parkinson’s disease. Neurophysiol Clin. 2008;38:459–65. 10.1016/j.neucli.2008.07.006. 19026965 10.1016/j.neucli.2008.07.006

[5] Bloem BR. Postural instability in Parkinson’s disease. Clin Neurol Neurosurg. 1992;94:41–5. 10.1016/0303-8467(92)90018-X. 10.1016/0303-8467(92)90018-x 1320515

[6] Doná F, Aquino CC, Gazzola JM, Borges V, Silva SMCA, Ganança FF, et al. Changes in postural control in patients with Parkinson’s disease: a posturographic study. Physiotherapy. 2016;102:272–9. 10.1016/j.physio.2015.08.009. 26582134 10.1016/j.physio.2015.08.009

[7] Grimbergen YA, Langston JW, Roos RA, Bloem BR. Postural instability in Parkinson’s disease: the adrenergic hypothesis and the locus coeruleus. Expert Rev Neurother. 2009;9:279–90. 10.1586/14737175.9.2.279. 19210201 10.1586/14737175.9.2.279

[8] Horak FB. Postural orientation and equilibrium: what do we need to know about neural control of balance to prevent falls? Age Ageing. 2006;35:ii7–11. 10.1093/ageing/afl077. 16926210 10.1093/ageing/afl077

[9] Kim SD, Allen NE, Canning CG, Fung VSC. Postural instability in patients with Parkinson’s disease: epidemiology, pathophysiology and management. CNS Drugs. 2013;27:97–112. 10.1007/s40263-012-0012-3. 23076544 10.1007/s40263-012-0012-3

[10] Hwang S, Agada P, Grill S, Kiemel T, Jeka JJ. A central processing sensory deficit with Parkinson’s disease. Exp Brain Res. 2016;234:2369–79. 10.1007/s00221-016-4642-4. 27059036 10.1007/s00221-016-4642-4 PMC4925186

[11] Siderowf A, McDermott M, Kieburtz K, Blindauer K, Plumb S, Shoulson I. Test–retest reliability of the unified Parkinson’s disease rating scale in patients with early Parkinson’s disease: results from a multicenter clinical trial. Mov Disord. 2002;17:758–63. 10.1002/mds.10011. 12210871 10.1002/mds.10011

[12] Landers MR, Backlund A, Davenport J, Fortune J, Schuerman S, Altenburger P. Postural instability in idiopathic Parkinson’s disease: discriminating fallers from nonfallers based on standardized clinical measures. J Neurol Phys Ther. 2008;32:56–61. 10.1097/NPT.0b013e3181761330. 18645292 10.1097/NPT.0b013e3181761330

[13] Goetz CG, Tilley BC, Shaftman SR, Stebbins GT, Fahn S, Martinez-Martin P, et al. Movement disorder society-sponsored revision of the unified Parkinson’s disease rating scale (MDS-UPDRS): scale presentation and clinimetric testing results. Mov Disord. 2008;23:2129–70. 10.1002/mds.22340. 19025984 10.1002/mds.22340

[14] Hoehn MM, Yahr MD. Parkinsonism: onset, progression, and mortality. Neurology. 1967;17:427–427. 10.1212/WNL.17.5.427. 6067254 10.1212/wnl.17.5.427

[15] Ebersbach G, Baas H, Csoti I, Müngersdorf M, Deuschl G. Scales in Parkinson’s disease. J Neurol. 2006;253:iv32–5. 10.1007/s00415-006-4008-0. 16944355 10.1007/s00415-006-4008-0

[16] Dibble LE, Lange M. Predicting falls in individuals with Parkinson disease: a reconsideration of clinical balance measures. J Neurol Phys Ther. 2006;30:60–7. 10.1097/01.NPT.0000282569.70920.dc. 16796770 10.1097/01.npt.0000282569.70920.dc

[17] Luque-Casado A, Novo-Ponte S, Sánchez-Molina JA, Sevilla-Sánchez M, Santos-Garciá D, Fernández-Del-Olmo M. Test–retest reliability of the timed up and go test in subjects with Parkinson’s disease: implications for longitudinal assessments. J Parkinsons Dis. 2021;11:2047–55. 10.3233/jpd-212687. 34334420 10.3233/JPD-212687

[18] Chastan N, Debono B, Maltête D, Weber J. Discordance between measured postural instability and absence of clinical symptoms in Parkinson’s disease patients in the early stages of the disease. Mov Disord. 2008;23:366–72. 10.1002/mds.21840. 18044726 10.1002/mds.21840

[19] Beuter A, Hernández R, Rigal R, Modolo J, Blanchet PJ. Postural sway and effect of levodopa in early Parkinson’s disease. Can J Neurol Sci. 2008;35:65–8. 10.1017/S0317167100007575. 18380279 10.1017/s0317167100007575

[20] Chen TZ, Xu GJ, Zhou GA, Wang JR, Chan P, Du YF. Postural sway in idiopathic rapid eye movement sleep behavior disorder: a potential marker of prodromal Parkinsons disease. Brain Res. 2014;1559:26–32. 10.1016/j.brainres.2014.02.040. 24602694 10.1016/j.brainres.2014.02.040

[21] Maetzler W, Mancini M, Liepelt-Scarfone I, Müller K, Becker C, van Lummel RC, et al. Impaired trunk stability in individuals at high risk for Parkinson’s disease. PLoS ONE. 2012;7: e32240. 10.1371/journal.pone.0032240. 22457713 10.1371/journal.pone.0032240 PMC3311622

[22] Curtze C, Nutt JG, Carlson-Kuhta P, Mancini M, Horak FB. Levodopa is a double-edged sword for balance and gait in people with Parkinson’s disease. Mov Disord. 2015;30:1361–70. 10.1002/mds.26269. 26095928 10.1002/mds.26269 PMC4755510

[23] Patel M, Nilsson MH, Rehncrona S, Tjernström F, Magnusson M, Johansson R, et al. Effects of deep brain stimulation on postural control in Parkinson’s disease. Comput Biol Med. 2020;122: 103828. 10.1016/j.compbiomed.2020.103828. 32658731 10.1016/j.compbiomed.2020.103828

[24] Nantel J, McDonald JC, Bronte-Stewart H. Effect of medication and STN-DBS on postural control in subjects with Parkinson’s disease. Parkinsonism Relat Disord. 2012;18:285–9. 10.1016/j.parkreldis.2011.11.005. 22130147 10.1016/j.parkreldis.2011.11.005

[25] Maurer C, Mergner T, Xie J, Faist M, Pollak P, Lucking CH. Effect of chronic bilateral subthalamic nucleus (STN) stimulation on postural control in Parkinson’s disease. Brain. 2003;126:1146–63. 10.1093/brain/awg100. 12690054 10.1093/brain/awg100

[26] Allen NE, Canning CG, Almeida LRS, Bloem BR, Keus SHJ, Löfgren N, et al. Interventions for preventing falls in Parkinson’s disease. Cochrane Database Syst Rev. 2022;6:CD011574. 10.1002/14651858.CD011574.pub2. 35665915 10.1002/14651858.CD011574.pub2 PMC9169540

[27] Hubble RP, Silburn PA, Naughton GA, Cole MH. Assessing stability in mild and moderate Parkinson’s disease: can clinical measures provide insight? Gait Posture. 2016;49:7–13. 10.1016/j.gaitpost.2016.06.002. 27348819 10.1016/j.gaitpost.2016.06.002

[28] Hasegawa N, Shah VV, Carlson-Kuhta P, Nutt JG, Horak FB, Mancini M. How to select balance measures sensitive to Parkinson’s disease from body-worn inertial sensors—separating the trees from the forest. Sensors. 2019;19:3320. 10.3390/s19153320. 31357742 10.3390/s19153320 PMC6696209

[29] Stylianou AP, McVey MA, Lyons KE, Pahwa R, Luchies CW. Postural sway in patients with mild to moderate Parkinson’s disease. Int J Neurosci. 2011;121:614–21. 10.3109/00207454.2011.602807. 21740307 10.3109/00207454.2011.602807

[30] Horak FB, Mancini M. Objective biomarkers of balance and gait for Parkinson’s disease using body-worn sensors. Mov Disord. 2013;28:1544–51. 10.1002/mds.25684. 24132842 10.1002/mds.25684 PMC3927718

[31] Ge W, Lueck CJ, Apthorp D, Suominen H. Which features of postural sway are effective in distinguishing Parkinson’s disease from controls? A systematic review. Brain Behav. 2021;11: e01929. 10.1002/brb3.1929. 33145991 10.1002/brb3.1929 PMC7821610

[32] Kamieniarz A, Michalska J, Marszałek W, Stania M, Słomka KJ, Gorzkowska A, et al. Detection of postural control in early Parkinson’s disease: clinical testing vs. modulation of center of pressure. PLoS ONE. 2021;16:1–12. 10.1371/journal.pone.0245353. 10.1371/journal.pone.0245353 PMC7802937 33434235

[33] Mancini M, Salarian A, Carlson-Kuhta P, Zampieri C, King L, Chiari L, et al. ISway: a sensitive, valid and reliable measure of postural control. J Neuroeng Rehabil. 2012;9:59. 10.1186/1743-0003-9-59. 22913719 10.1186/1743-0003-9-59 PMC3481400

[34] Mancini M, Horak FB, Zampieri C, Carlson-Kuhta P, Nutt JG, Chiari L. Trunk accelerometry reveals postural instability in untreated Parkinson’s disease. Parkinsonism Relat Disord. 2011;17:557–62. 10.1016/j.parkreldis.2011.05.010. 21641263 10.1016/j.parkreldis.2011.05.010 PMC5327861

[35] Rocchi L, Chiari L, Horak FB. Effects of deep brain stimulation and levodopa on postural sway in Parkinson’s disease. J Neurol Neurosurg Psychiatry. 2002;73:267–74. 10.1136/jnnp.73.3.267. 12185157 10.1136/jnnp.73.3.267 PMC1738049

[36] Beretta VS, Barbieri FA, Orcioli-Silva D, dos Santos PCR, Simieli L, Vitório R, et al. Can postural control asymmetry predict falls in people with Parkinson’s disease? Mot Control. 2018;22:449–61. 10.1123/mc.2017-0033. 10.1123/mc.2017-0033 29651890

[37] Curtze C, Nutt JG, Carlson-Kuhta P, Mancini M, Horak FB. Objective gait and balance impairments relate to balance confidence and perceived mobility in people with Parkinson disease. Phys Ther. 2016;96:1734–43. 10.2522/ptj.20150662. 27149959 10.2522/ptj.20150662 PMC5088223

[38] Kamieniarz A, Michalska J, Brachman A, Pawłowski M, Słomka KJ, Juras G. A posturographic procedure assessing balance disorders in Parkinson’s disease: a systematic review. Clin Interv Aging. 2018;13:2301–16. 10.2147/CIA.S180894. 30519012 10.2147/CIA.S180894 PMC6237244

[39] Dotchin C, Walker R. The management of Parkinson’s disease in sub-Saharan Africa. Expert Rev Neurother. 2012;12:661–6. 10.1586/ern.12.52. 22650168 10.1586/ern.12.52

[40] Mei J, Desrosiers C, Frasnelli J. Machine learning for the diagnosis of Parkinson’s disease: a review of literature. Front Aging Neurosci. 2021;13:1–41. 10.3389/fnagi.2021.633752. 10.3389/fnagi.2021.633752 PMC8134676 34025389

[41] Li Y, Zhang S, Odeh C. Automated classification of postural control for individuals with Parkinson’s disease using a machine learning approach: a preliminary study. J Appl Biomech. 2020;36:334–9. 10.1123/JAB.2019-0400. 32736341 10.1123/jab.2019-0400

[42] Fadil R, Huether A, Brunnemer R, Blaber AP, Lou JS, Tavakolian K. Early detection of Parkinson’s disease using center of pressure data and machine learning. In: Proceedings of the annual international conference of the IEEE engineering in medicine and biology society (EMBS), Mexico, 2021, p. 2433–6. 10.1109/EMBC46164.2021.9630451. 10.1109/EMBC46164.2021.9630451 34891772

[43] Lecun Y, Bengio Y, Hinton G. Deep learning. Nature. 2015;521:436–44. 10.1038/nature14539. 26017442 10.1038/nature14539

[44] Krizhevsky A, Sutskever I, Hinton GE. ImageNet classification with deep convolutional neural networks. Commun ACM. 2017;60:84–90. 10.1145/3065386.

[45] Loh HW, Hong W, Ooi CP, Chakraborty S, Barua PD, Deo RC, et al. Application of deep learning models for automated identification of Parkinson’s disease: a review (2011–2021). Sensors. 2021;21:1–25. 10.3390/s21217034 PMC8587636 34770340

[46] Engel D, Schwenk JCB, Schütz A, Morris AP, Bremmer F. Multi-segment phase coupling to oscillatory visual drive. Gait Posture. 2021;86:132–8. 10.1016/j.gaitpost.2021.03.010. 33721690 10.1016/j.gaitpost.2021.03.010

[47] Engel D, Student J, Schwenk JCB, Morris AP, Waldthaler J, Timmermann L, et al. Visual perturbation of balance suggests impaired motor control but intact visuomotor processing in Parkinson’s disease. J Neurophysiol. 2021;126:1076–89. 10.1152/jn.00183.2021. 34469704 10.1152/jn.00183.2021

[48] Student J, Engel D, Timmermann L, Bremmer F, Waldthaler J. Visual perturbation suggests increased effort to maintain balance in early stages of Parkinson’s to be an effect of age rather than disease. Front Hum Neurosci. 2022;16: 762380. 10.3389/fnhum.2022.762380. 35308620 10.3389/fnhum.2022.762380 PMC8924037

[49] Postuma RB, Berg D, Stern M, Poewe W, Olanow CW, Oertel W, et al. MDS clinical diagnostic criteria for Parkinson’s disease. Mov Disord. 2015;30:1591–601. 10.1002/mds.26424. 26474316 10.1002/mds.26424

[50] Ciesielska N, Sokołowski R, Mazur E, Podhorecka M, Polak-Szabela A, Kędziora-Kornatowska K. Is the Montreal cognitive assessment (MoCA) test better suited than the mini-mental state examination (MMSE) in mild cognitive impairment (MCI) detection among people aged over 60? Meta-analysis. Psychiatr Pol. 2016;50:1039–52. 10.12740/PP/45368. 27992895 10.12740/PP/45368

[51] Winter DA. Biomechanics and motor control of human movement. New York: Wiley; 2009.

[52] LeNail A. NN-SVG: publication-ready neural network architecture schematics. J Open Source Softw. 2019;4:747. 10.21105/joss.00747.

[53] Paszke A, Gross S, Massa F, Lerer A, Bradbury J, Chanan G, et al. PyTorch: an imperative style, high-performance deep learning library. arXiv:1912.01703. 2019. 10.48550/arXiv.1912.01703.

[54] Rainio O, Teuho J, Klén R. Evaluation metrics and statistical tests for machine learning. Sci Rep. 2024;14:6086. 10.1038/s41598-024-56706-x. 38480847 10.1038/s41598-024-56706-x PMC10937649

[55] Selvaraju RR, Cogswell M, Das A, Vedantam R, Parikh D, Batra D. Grad-CAM: visual explanations from deep networks via gradient-based localization. Int J Comput Vis. 2020;128:336–59. 10.1007/s11263-019-01228-7.

[56] Dhore V, Bhat A, Nerlekar V, Chavhan K, Umare A. Enhancing explainable AI: a hybrid approach combining GradCAM and LRP for CNN interpretability. arXiv:2405.12175. 2024.

[57] Richmond SB, Fling BW, Lee H, Peterson DS. The assessment of center of mass and center of pressure during quiet stance: current applications and future directions. J Biomech. 2021;123: 110485. 10.1016/j.jbiomech.2021.110485. 34004395 10.1016/j.jbiomech.2021.110485

[58] Horak FB, Macpherson JM. Postural orientation and equilibrium. In: Comprehensive physiology. New York: Wiley; 1996. p. 255–92.

[59] Peterka RJ. Sensorimotor integration in human postural control. J Neurophysiol. 2002;88:1097–118. 10.1152/jn.2002.88.3.1097. 12205132 10.1152/jn.2002.88.3.1097

[60] Winter DA, Patla AE, Prince F, Ishac M, Gielo-Perczak K. Stiffness control of balance in quiet standing. J Neurophysiol. 1998;80:1211–21. 10.1152/jn.1998.80.3.1211. 9744933 10.1152/jn.1998.80.3.1211

[61] Djaldetti R, Ziv I, Melamed E. The mystery of motor asymmetry in Parkinson’s disease. Lancet Neurol. 2006;5:796–802. 10.1016/S1474-4422(06)70549-X. 16914408 10.1016/S1474-4422(06)70549-X

[62] Riederer P, Jellinger KA, Kolber P, Hipp G, Sian-Hülsmann J, Krüger R. Lateralisation in Parkinson disease. Cell Tissue Res. 2018;373:297–312. 10.1007/s00441-018-2832-z. 29656343 10.1007/s00441-018-2832-z

[63] Mitchell SL, Collin JJ, De Luca CJ, Burrows A, Lipsitz LA. Open-loop and closed-loop postural control mechanisms in Parkinson’s disease: increased mediolateral activity during quiet standing. Neurosci Lett. 1995;197:133–6. 10.1016/0304-3940(95)11924-L. 8552278 10.1016/0304-3940(95)11924-l

[64] Błaszczyk JW, Orawiec R. Assessment of postural control in patients with Parkinson’s disease: sway ratio analysis. Hum Mov Sci. 2011;30:396–404. 10.1016/j.humov.2010.07.017. 20800915 10.1016/j.humov.2010.07.017

[65] Błaszczyk JW, Orawiec R, Duda-Kłodowska D, Opala G. Assessment of postural instability in patients with Parkinson’s disease. Exp Brain Res. 2007;183:107–14. 10.1007/s00221-007-1024-y. 17609881 10.1007/s00221-007-1024-y

[66] Rocchi L, Chiari L, Cappello A, Horak FB. Identification of distinct characteristics of postural sway in Parkinson’s disease: a feature selection procedure based on principal component analysis. Neurosci Lett. 2006;394:140–5. 10.1016/j.neulet.2005.10.020. 16269212 10.1016/j.neulet.2005.10.020

[67] Sebastia-Amat S, Tortosa-Martínez J, Pueo B. The use of the static posturography to assess balance performance in a Parkinson’s disease population. Int J Environ Res Public Health. 2023;20:981. 10.3390/ijerph20020981. 36673738 10.3390/ijerph20020981 PMC9859212

[68] da Conceição NR, de Sousa PN, Pereira MP, Gobbi LTB, Vitório R. Utility of center of pressure measures during obstacle crossing in prediction of fall risk in people with Parkinson’s disease. Hum Mov Sci. 2019;66:1–8. 10.1016/j.humov.2019.03.010. 30889495 10.1016/j.humov.2019.03.010

[69] Warnica MJ, Weaver TB, Prentice SD, Laing AC. The influence of ankle muscle activation on postural sway during quiet stance. Gait Posture. 2014;39:1115–21. 10.1016/j.gaitpost.2014.01.019. 24613374 10.1016/j.gaitpost.2014.01.019

[70] Paillard T, Noé F. Techniques and methods for testing the postural function in healthy and pathological subjects. Biomed Res Int. 2015;2015: 891390. 10.1155/2015/891390. 26640800 10.1155/2015/891390 PMC4659957

[71] Wu T, Hallett M. The cerebellum in Parkinson’s disease. Brain. 2013;136:696–709. 10.1093/brain/aws360. 23404337 10.1093/brain/aws360 PMC7273201

[72] Mirdamadi JL. Cerebellar role in Parkinson’s disease. J Neurophysiol. 2016;116:917–9. 10.1152/jn.01132.2015. 26792889 10.1152/jn.01132.2015 PMC5009206

[73] Abbruzzese G, Berardelli A. Sensorimotor integration in movement disorders. Mov Disord. 2003;18:231–40. 10.1002/mds.10327. 12621626 10.1002/mds.10327

[74] Jacobs JV, Horak FB. Abnormal proprioceptive-motor integration contributes to hypometric postural responses of subjects with Parkinson’s disease. Neuroscience. 2006;141:999–1009. 10.1016/j.neuroscience.2006.04.014. 16713110 10.1016/j.neuroscience.2006.04.014

[75] Rivlin-Etzion M, Marmor O, Heimer G, Raz A, Nini A, Bergman H. Basal ganglia oscillations and pathophysiology of movement disorders. Curr Opin Neurobiol. 2006;16:629–37. 10.1016/j.conb.2006.10.002. 17084615 10.1016/j.conb.2006.10.002

[76] Prange S, Danaila T, Laurencin C, Caire C, Metereau E, Merle H, et al. Age and time course of long-term motor and nonmotor complications in Parkinson disease. Neurology. 2019;92:E148–60. 10.1212/WNL.0000000000006737. 30541866 10.1212/WNL.0000000000006737

[77] Gago MF, Fernandes V, Ferreira J, Silva H, Rodrigues ML, Rocha L, et al. The effect of levodopa on postural stability evaluated by wearable inertial measurement units for idiopathic and vascular Parkinson’s disease. Gait Posture. 2015;41:459–64. 10.1016/j.gaitpost.2014.11.008. 25480163 10.1016/j.gaitpost.2014.11.008

[78] Holford N, Nutt JG. Disease progression, drug action and Parkinson’s disease: why time cannot be ignored. Eur J Clin Pharmacol. 2008;64:207–16. 10.1007/s00228-007-0427-9. 18092155 10.1007/s00228-007-0427-9 PMC3390311

[79] Sarkar S, Raymick J, Imam S. Neuroprotective and therapeutic strategies against Parkinson’s disease: recent perspectives. Int J Mol Sci. 2016;17:904. 10.3390/ijms17060904. 27338353 10.3390/ijms17060904 PMC4926438

